# Geriatricians and occupational therapists’ perspectives on the role of occupational therapy in delirium care in long-term care settings: a multi-site study

**DOI:** 10.1007/s41999-025-01282-0

**Published:** 2025-08-05

**Authors:** Christian Pozzi, Giulia Borgonovo, Juan Antonio Lopez Segura, ABirgitta Gunnarsson, Laura N. Gitlin

**Affiliations:** 1 Centre of Competence on Ageing, University of Applied Sciences and Arts of Southern Switzerland (SUPSI), Manno, Switzerland; 2 ErgoSUM Switzerland, Tesserete, Switzerland; 3 Parc Sanitari Pere Virgili, Barcelona, Spain; 4 Institute of Neuroscience and Physiology, Department of Health and Rehabilitation, University of Gothenburg, Gothenburg, Sweden; 5 Department of Research and Development, Region Kronoberg, Växjö, Sweden; 6 College of Nursing and Health Professions, Drexel University, Philadelphia, PA USA

**Keywords:** Interprofessional collaboration, Delirium care, Qualitative research, Rehabilitation, Occupational therapy

## Abstract

**Background:**

Delirium is a frequent and serious condition in older adults in long-term care (LTC), often underdiagnosed and insufficiently addressed despite its significant impact on morbidity and mortality. Occupational therapists (OTs) may play a key role in non-pharmacological prevention and treatment approaches, yet their specific contribution may remain unclear and undervalued in clinical practice.

**Objective:**

This study explored the perceptions of geriatricians and OTs regarding the role of OT in delirium care in LTC settings across three European countries.

**Methods:**

A qualitative, multi-site study was conducted through semi-structured interviews with six geriatrician–occupational therapist dyads working in LTC settings in Italy, Spain, and Switzerland. Transcripts were analyzed thematically through inductive coding.

**Results:**

Three main conceptual categories emerged: (1) the perceptions of the OT’s role by geriatricians, (2) the goals and interventions of OTs in delirium care, and (3) barriers and facilitators to OT involvement. Geriatricians with greater familiarity with OTs recognized their role in prevention through environmental modifications and meaningful activity promotion, whereas others saw OTs’ contributions as overlapping with physiotherapy. OTs often lacked standardized tools and specific training for delirium care, and caregiver involvement was minimal. Barriers included time constraints, unclear interprofessional roles, and limited geriatric training; facilitators included strong interdisciplinary collaboration and supportive teams.

**Conclusion:**

Enhancing the role of occupational therapy in delirium care requires clearer interprofessional definitions, dedicated training, and structural support in LTC. Structured collaboration between geriatricians and OTs may improve prevention and management outcomes for delirium in older adults.

**Supplementary Information:**

The online version contains supplementary material available at 10.1007/s41999-025-01282-0.

## Key summary points

- Occupational therapists (OTs) are increasingly involved in delirium care in long-term care (LTC) settings, yet their role remains variably understood and inconsistently integrated by geriatricians.
- OTs primarily contribute through patient-centered, non-pharmacological interventions aimed at enhancing function and quality of life, but their involvement in prevention and screening is less defined.
- Limited role clarity, time constraints, and poor interdisciplinary integration hinder effective OT participation in delirium care.
- Key facilitators include strong interprofessional collaboration and recognition of the patient's individual needs and preferences.
- Structured training and organizational models are needed to enhance the visibility and integration of OT in delirium prevention and management in LTC.

**Supplementary Information:**

The online version contains supplementary material available at 10.1007/s41999-025-01282-0.

## Introduction

Delirium is an acute and fluctuating geriatric syndrome, particularly common in hospitalized older adults, and associated with serious outcomes, such as increased mortality, functional decline, institutionalization, and risk of dementia [1, 2]. It remains underdiagnosed and complex, requiring an interdisciplinary approach to care due to its multifactorial pathophysiology [3].

Prevention is essential, as a significant portion of delirium episodes is considered preventable through early, targeted, non-pharmacological interventions [4]. These interventions should go beyond single-profession training, involving physicians, nurses, physiotherapists, and OTs, with the support of trained family members and volunteers [5, 6]. OTs are health professionals trained to promote autonomy, function, and well-being through engagement in meaningful activities. Their core competencies include functional assessment, cognitive and sensory stimulation, and environmental adaptation. In most European countries, occupational therapy education is delivered through a 3- to 4-year university program combining clinical training and theoretical coursework. In the context of delirium, OTs may contribute by supporting orientation, maintaining daily routines, and enhancing participation through non-pharmacological interventions tailored to the individual’s cognitive and functional profile [7]. Key strategies include reorientation, medication review, sleep–wake cycle regulation, early mobilization, proper hydration and nutrition, and sensory aid use [8, 9]. Collaboration and structured communication between professionals are crucial for effective care [10, 11].

Geriatricians and OTs play key roles in delirium care, with the former focusing on diagnosis and integrated treatment, and the latter on functional recovery through daily activities [12–14]. However, the extent to which geriatricians acknowledge the role of OT in this area remains unclear, as does the optimal therapeutic approach by OTs for people with delirium [15].

Greater mutual understanding is vital to support the growing evidence on multimodal, team-based non-pharmacological interventions [16]. This study explores how geriatricians perceive the role of OT in delirium care, including the assessments conducted, how goals are set, and what interventions OTs implement in LTC.

## Materials and methods

A qualitative research design [17] was used, employing a structured questionnaire (Supplementary Materials) that included the specific questions administered to both occupational therapists and geriatricians. These questions differed between the two professional groups to better explore their respective perspectives on occupational therapy for people with delirium in LTC settings. The interviews were conducted via Microsoft Teams. Topics addressed demographic data, reasons for referral to OT, the role of OTs in delirium prevention and treatment, and the collaboration between OTs and other healthcare professionals. The interviews also explored barriers and facilitators to effective OT involvement in delirium care and potential improvements to OT services in geriatric settings. Six online qualitative interviews were conducted with six stable geriatrician—OTs dyads working in LTC settings. The term “stable dyads” refers to professionals who had been working collaboratively for at least 6 months within the same interdisciplinary LTC team, ensuring continuity and a shared clinical context. The interviews were carried out by OTs with proven clinical research competence in geriatrics and psychogeriatrics (holding either a Master of Science, or a Ph.D.). The interviews were conducted in Italian or English, based on participant preference, and focused on the geriatrician–OT collaboration and the occupational therapy process in delirium prevention and care.

Interviews took place in the participants’ usual clinical environments (OT offices or medical consulting rooms). To ensure privacy and avoid response contamination, each professional was interviewed individually. At the beginning of the interview, participants were welcomed with a brief research introduction, followed by a collection of biographical data and the signing of informed consent. The geriatrician was interviewed first, followed by the OT. Each individual interview was recorded and lasted between 30 and 75 min.

### Participants and setting

The study participants were geriatricians working in LTC settings where at least one OT was present in the interdisciplinary team and potentially involved in the prevention and/or treatment of individuals with delirium. A purposive sampling strategy was employed to select key participants directly engaged in the topic under investigation. This approach allowed for the collection of informed and detailed perspectives, essential for a qualitative exploration of the phenomenon.

The inclusion criteria were as follows:

(A) Geriatricians working in LTC in Italy, Switzerland, or Spain.
(B) OTs working in LTC in Italy, Switzerland, or Spain.
(C) Provision of informed consent to participate in the study.

The exclusion criteria included:

1. Geriatricians who do not work with OTs as part of the interprofessional team or do not manage patients with delirium

### Data analysis

The analysis was conducted in a systematic and iterative process [18]. Initially, the transcripts of the interviews were thoroughly reviewed to gain a deep understanding of the participants' responses. Following this, the data were organized and categorized into meaningful segments, allowing for the identification of recurring themes. These themes were then refined and analyzed to better understand the key factors that influence the role of OTs in delirium care. Using this approach, the study ensured a clear and structured interpretation of the data, with the aim of revealing the most significant insights from the participants' perspectives.

Qualitative data analysis followed a systematic multi-phase process. First, interviews were fully transcribed to ensure accurate representation of participant responses. The transcripts were then independently reviewed by both interviewers. Each researcher individually identified relevant themes. In the second phase, an inductive analysis was used to build conceptual categories. Relevant text segments were coded using descriptive labels that reflected the implicit and explicit meaning of responses. These codes were then grouped into broader categories representing recurring and emerging themes. Researcher triangulation was employed to ensure reliability and validity through comparison and discussion of the coding, resolving interpretive discrepancies collaboratively. Finally, conceptual categories emerged, and were reflected and discussed, until consensus was reached.

### Ethics

The study was conducted in accordance with the principles outlined in the Declaration of Helsinki (World Medical Association, 2013). All participants provided their informed consent before participation, ensuring ethical compliance.

## Results

The total sample consisted of six geriatricians and six OTs. Demographic information, professional background, and country of practice are detailed in Table 1 (geriatricians) and Table 2 (OTs).

**Table 1:** Characteristics of the sample of interviewed geriatricians

Sample 1 Geriatricians	*N* = 6
Country – Italy – Spain – Switzerland	*n* = 2 *n* = 2 *n* = 2
Sex (W)	*n* = 5
Age (years), median (IQR)	49.5 (45—54)
Experience (years) – From 1 to 10 – From 11 to 20 – More than 20	*n* = 0 *n* = 3 *n* = 3
Experience (years) in LTC – From 1 to 10 – From 11 to 20 – More than 20	*n* = 5 *n* = 1 *n* = 0
Number of beds available in LTC – From 0 to 30 – From 31 to 60 – From 61 to 80	n = 2 n = 1 n = 3
Knowledge of the therapeutic process in occupational therapy (assessment, goal setting, treatment) – Yes – No	*n* = 4 *n* = 2
Knowledge of the therapeutic process in occupational therapy for individuals with delirium (assessment, goal setting, treatment) – Yes – No	*n* = 2 *n* = 4

**Table 2:** Characteristics of the sample of interviewed OTs

Sample 2 Occupational therapists	*N* = 6
Country – Italy – Spain – Switzerland	*n* = 2 *n* = 2 *n* = 2
Sex (W)	*n* = 5
Age (years), median (IQR)	33 (30–35)
Experience (years) – From 1 to 10 – From 11 to 20 – More than 20	*n* = 5 *n* = 1 *n* = 0
Experience (years) in LTC – From 1 to 3 – From 4 to 10 – More than 10	*n* = 2 *n* = 3 *n* = 1
Number of occupational therapists in the service – 1 – 2 to 3 – More than 3	*n* = 3 *n* = 3 *n* = 0
Occupational therapist-to-bed ratio	1 OT per 32 beds ratio
During your university studies, did you receive lectures related to occupational therapy and delirium – Yes – No	*n* = 2 *n* = 4
After graduation, have you attended conferences, seminars, or webinars related to occupational therapy and delirium? – Yes – No	*n* = 2 *n* = 4

The total sample of the study was geographically divided equally between Italy, Spain, and Switzerland. Most participants were female (*n* = 10). The geriatricians had a median age of 49.5 years with varied work experience. Three of them had between 11 and 20 years of experience, and the remaining of them had over 20 years. However, their experiences of LTC were primarily between 1 and 10 years (n = 5). Regarding knowledge of the OT process, geriatricians reported being informed, while only two of them had specific knowledge about the application of the OT process in the care of individuals with delirium.

As for the OTs interviewed, they had a median age of 33 years, and five of them had between 1 and 10 years of work experience. Their experiences in LTC varied more, with three of them having between 4 and 10 years of experience. Knowledge of the therapeutic process in the care of individuals with delirium was limited, with only two of them having received specific training during their university education, and only two of them having attended courses or conferences addressing this topic.

The inductive qualitative survey allowed us to outline three conceptual categories. Within each category, we coded relevant text segments representing recurring and emerging themes. Each sub-category was named by the researchers to clarify and make accessible the implicit and explicit meaning found in the interviewees' responses.

(D) **Conceptual category 1: Tasks and duties assigned by geriatricians to the OT service in LTC**

The views of geriatricians on the role and competencies of OT differ significantly and are largely influenced by their level of knowledge about OT as a profession. Geriatricians highlighted a direct correlation between knowledge of OT and the ability to define a specific and distinct role for it in the context of LTC and delirium care.

Geriatricians with an in-depth understanding of OT as a rehabilitative healthcare profession recognize the OT’s fundamental contribution to delirium prevention through targeted strategies, such as environmental adaptation, the use of assistive devices, and the promotion of meaningful activities for patients with delirium. In treatment, they attribute to OT an active role in interaction with the caregiver, effective communication with the patient, and staff training.

On the other hand, limited knowledge of OT leads to a less-defined view, often overlapping with other healthcare professions, reducing OT to a generic intervention for functional recovery post-delirium, in some cases confused with physiotherapy approaches.

This variability in the perception of OT has significant implications for the promotion of the profession and its integration into delirium care protocols, as reflected in differing future visions for OT’s role.

1. **Geriatricians’ understanding of occupational therapy**

Comprehensive understanding

Several participants expressed this perspective, illustrated by the quote:

In university, during my specialty in geriatrics, I had never heard of it. But honestly, we never heard about physiotherapy either. I found this OT in the ward, and now I can't do without them. I understood it through clinical cases and, above all, by listening to patients who are happy to undergo OT: especially the more fragile ones feel understood. So, I don't know if I know OT, but I know and listen to the patients who receive OT, and it seems like a fantastic profession

Limited understanding

Other participants reported uncertainty regarding distinctions among professionals:

Honestly, I don’t really know the differences between the various professionals: for example, I think physiotherapists and OTs do things... maybe not the same, but similar

Between physiotherapists, educators, and OTs, I still don't understand the peculiarities of one profession or the other: all are very skilled, but the philosophical differences behind the profession are not very clear to me as a doctor

(A2) **Geriatricians’ perceptions of OT tasks in delirium care**

Defined tasks associated WITG comprehensive OT knowledge

Participants with detailed understanding attributed specific roles: *“I firmly believe that OTs possess the appropriate cultural and clinical competencies to be the key rehabilitation professionals for both the prevention and treatment of delirium. I am especially convinced about prevention: using prosthetics, adapting the environment, promoting movement and meaningful occupation. Regarding treatment, it is crucial to involve the caregiver during rehabilitative sessions, apply effective communication skills, and train nursing and care staff.”*

Tailor-made with specific activities: also, during the delirium experience. Non-pharmacological activities should not be abandoned during delirium. The OT can manage it.

Generic tasks linked to limited to knowledge

Other views included: *“First, the goal is to return the patient to their pre-delirium state, which often seems impossible. After that, we resume the same activities our physiotherapists carry out. I believe the main objective is to restore the pre-delirium condition and then reschedule OT accordingly.”*

And then of course there is the pharmacological intervention. Afterwards, we assess how much functional recovery has occurred and accordingly reprogram OT.

(A3) **Geriatricians’ expectations for the future role of OT in delirium care**

Specific professional roles supported by in-depth knowledge. Many participants reflected similar views, for example:

The OT inspires great trust in people with delirium, demonstrates strong empathy, and possesses a remarkable sensitivity in understanding patient emotions. Furthermore, they communicate with healthcare workers and other team members, explaining what needs to be done and how.

Training the team: currently, I see OTs working wonderfully with sensitive and capable colleagues but struggling to be understood by those less predisposed to empathy. Yet, both types exist within every team, and OTs must be able to work effectively with both.

Generalized role linked to limited knowledge. A diverging opinion came from this respondent:

In this context, occupation may offer a sense of hope and future. It’s difficult to quantify in patients, but I believe it alleviates some of the depressive burden that often accompanies these patients and situations.

(E) **Conceptual category 2: Assessment, goals, and interventions of OTs in the prevention and treatment of individuals with delirium**

The prevention and early identification of delirium, the definition of targeted therapeutic goals, and the implementation of specific interventions were the key aspects we aimed to explore to better define the role of OTs in managing this syndrome within LTC settings. Through the analysis of qualitative responses provided by OTs, three key domains of their clinical practice emerged:

- strategies for prevention and delirium screening/assessment tools;
- goals guiding OT interventions in patients with delirium;
- OT interventions implemented with individuals experiencing delirium.

The participants’ experiences revealed a wide range of approaches and practices. Prevention appeared to be a less clearly defined phase for OTs, as it is often embedded in routine OT interventions. On the other hand, therapeutic actions focused primarily on enhancing physical and cognitive abilities during the delirium episode, with an emphasis on simple activities and targeted environmental stimulation.

A variety of screening and assessment practices also emerged. Some OTs highlighted the importance of daily evaluation of cognitive changes, while others perceived delirium assessment as a function integrated with other team members.

Below are the most representative quotes collected during the qualitative analysis, coded according to prevention and treatment.

1. **Prevention strategies and screening/assessment tools for delirium**

Delirium prevention and OT

Being proactive, engaging the patient by facilitating their activities. That’s my guiding principle.

First, it is important to focus on the environment and ensure that the person can remain active and maintain their routines. I place their name on the door, minimize environmental overload, promote spaces with natural light, protect the environment to reduce risks, and simplify routines.

Screening/assessment tools for delirium

*Specific for delirium:*

We don’t use a specific screening tool in every treatment for delirium: if I notice something wrong with the patient, I ask the nurse or the attending physician to come and assess the situation. The facility is small, and I think that helps us. I don’t believe that introducing a formal delirium screening test would be beneficial. However, in the case of cognitive fluctuations, I immediately contact the caregiver by phone or speak with them during visiting hours to understand whether this change was already present at home — a sort of triage to distinguish between delirium and Behavioral and Psychological Symptoms of Dementia (BPSD).

*Non-specific for delirium*:

When we notice through observation that something is not right, we perform a Mini-Mental State Examination to check for cognitive decline.

Our assessments are not focused on delirium. When we conduct evaluations, we assess cognitive functioning, activity levels, and interests in a general sense.

No delirium-specific tools — I evaluate life history and use the Allen Cognitive Test. Of course, if there’s a clear cognitive change, I alert the doctor because it might be delirium, but I don’t think the Allen Cognitive Test is specific for delirium.

We don’t have specific tests to carry out daily for delirium. In my view, this should become part of an institutional protocol, not something left to the individual initiative of the OT.

(B2) **Goals guiding OT interventions in person with delirium**

Aimed at the person with delirium

Our aim is to keep the person active during the delirium episode to prevent disability: we take a proactive approach, encouraging both the person and the caregiver to maintain participation or autonomy in ADLs, for example.

Maintaining cognitive abilities: if the patient already has dementia, we try to preserve their cognitive functioning after the delirium episode.

Working in a team to ensure that the delirium episode is brief in both duration and intensity.

Aimed at the caregiver

No OT treatment goals specifically aimed at the caregiver were reported.

(B3) **OT interventions with person with delirium**

OT and person with delirium-specific intervention

Certainly, a quieter environment, with fewer stimuli, simplified activities—ideally very basic daily life tasks—promoting relaxation and well-being, without requiring too much cognitive effort.

Engagement of the person with delirium must be maximized; we do not propose exercises, but rather simple everyday activities.

In the acute phase of delirium: natural light, protect the environment, not to be risky for the person, simplify routines.

OT and person with delirium-non-specific intervention

Certainly, if the patient is in an advanced state (advanced dementia), we focus on sensory stimulation—visual, tactile—using for instance music therapy, cinema therapy, and also stimulating fine motor skills and tactile sensitivity.

Daily activities that are suitable, structured, and specific to the person.

The intervention is quite non-specific and poorly aligned with the person’s personality. Let’s say we adapt what we should be doing since the person is experiencing delirium. For example: if someone who underwent spinal surgery is in delirium, we still begin with transfers and resumption of ADLs, but we certainly adjust our expectations considering delirium. That said, we currently do not involve the caregiver as we should.

OT and the caregiver of the person with delirium

If the physician tells me the person has delirium and I happen to meet a family member during the visit, I inform them and provide some guidance.

It would be great to share information with the caregiver, but it’s not always possible: meetings usually take place during family visiting hours. It would be nice, though.

(F) **Conceptual category 3: barriers and facilitators of the implementation of OT in the prevention and care of person with delirium.**

The perceptions of OTs regarding the conditions that hinder or, on the contrary, facilitate the implementation of OT services in LTC for the care of people with delirium have been explored. Specifically, during the interviews, we asked participants to reflect on resources, structural and organizational limitations, interprofessional dynamics, as well as aspects related to training and professional culture that influence the ability to intervene effectively in favor of individuals with delirium.

This section allows us to collect the main barriers and facilitators identified through thematic analysis, offering a realistic and contextualized view of the potential and challenges OTs face in promoting a proactive and person-centered approach to delirium management.

1. **Facilitators**

The interdisciplinary team

The team supports me a lot, and when I don’t understand some variables of delirium, they help me to better understand them. The work environment makes things much easier for me.

I’ve been here for a year. When I arrived, I had to learn a lot, so my team guided me, and now I feel that I have many more skills, even in analyzing different situations.

The meetings with the team allow me to stay up to date on the situation; I learn a lot from these meetings by listening to those who have more experience than me on delirium.

Having the clinical picture under control helps and facilitates me: this way I can educate and inform everyone (family members, staff, etc.) and I feel more at ease.

The ability to understand the expectations and wishes of the patient: in my opinion, this is the key that would also improve the care of delirium.

(C2) **Barriers**

Time

Having more time and more weekly sessions for fragile individuals to prevent delirium through meaningful activities.

The environment

Same space with physio, there isn’t an OT room for treatment. One is the hospital environment with few meaningful objects for that patient.

The interdisciplinary team

The team shouldn’t think that the OT ‘keeps them busy.’ Carrying out activities is a task for the whole team, not just the OT, who can certainly help in selecting the right activities. Furthermore, it’s important to work on the concept of rehabilitation in geriatrics: ageism is still very present.

Clarity of role… in theory, everything is clear for the team, but it’s not like that. Working interdisciplinarity is difficult. It’s hard to understand the role because there’s a lack of capacity to work together. And maybe the patient and their wishes aren’t really central to the care process.

Experience and self-perception of competence

Certainly, I have little experience; I see people with delirium sporadically. Maybe I should gain experience in other settings where there are more delirium episodes. But I don’t know… I need to think about it. It’s not easy for an OT to see themselves as an expert in delirium.

Certainly, the OT lacks competence in this area. And, in my opinion, this doesn’t allow us to be really active in the team’s training. To be a leader for these patients, you need to be very competent, and today, in my opinion, we aren’t (Table 3).

**Table 3:** Summary of main findings by domain

Domain	Emerging themes	Barriers	Facilitators
Perception of OT role	Variable understanding of OT’s scope; low professional visibility	Lack of OT knowledge among geriatricians; role confusion with other professionals	Direct clinical exposure to OT; positive patient feedback
OT practice in delirium care	Predominantly post-delirium interventions; prevention less structured	Absence of shared protocols and screening tools	Meaningful, activity-based engagement; tailored interventions
Interprofessional collaboration	Collaboration valued but inconsistently structured	Time constraints; lack of training in team dynamics	Team support; shared clinical goals; interprofessional education

## Discussion

The findings of the study show variability in the perception of the OTs’ role among geriatricians in the management and prevention of delirium in LTC settings. Participants' narratives suggest that knowledge of the OT contribution to delirium management and prevention is closely related to direct experience with such professionals and the prior training received. Geriatricians with greater familiarity with OT tend to recognize its specific value in prevention through targeted interventions on the environment, functional activation, and cognitive stimulation. In contrast, limited knowledge seems to foster role overlaps with other professionals, reducing OT to a generic support for motor rehabilitation. This partial understanding of the role aligns with findings in the literature, which highlight persistent difficulties in fully integrating OT into interdisciplinary teams, partly due to the lack of recognition of the discipline in training and organizational pathways in healthcare settings [5, 16, 19]. The lack of awareness of the preventive and therapeutic value of OT could represent a barrier to the effective treatment of delirium, with implications for the overall quality of care provided [20].

The collected data suggest that, while recognizing the clinical importance of delirium management, many geriatricians involved in the study tend not to explicitly define a specific role for OTs within the pathways adopted in LTC facilities. This ambiguity may reflect a partial or fragmented understanding of the general competencies of OTs, especially where there is a lack of solid experience in the geriatric context.

As highlighted by the collected experiences, the limited years of service in LTC facilities (1–10 years for most of the sample) may contribute to a less-defined and recognized professional identity. The absence of professionals with long-standing experience in the team may constitute a barrier to developing a shared professional identity. The average age of the participating OTs, which is 33, suggests the presence of a generation of professionals in the early stages of their clinical careers. The narratives reveal strong motivation and theoretical updating consistent with the latest evidence, aspects that may represent added value in multidisciplinary contexts. However, this professional profile might face integration difficulties in established teams, where clinical experience and role dynamics are already strongly structured. The findings of this study, while referring to a limited sample, raise questions about the process of building the OT professional identity in interprofessional contexts. This prompts reflection, in line with Darawsheh [20] on the need to facilitate reflective practices and collaboration models that can enhance the specific contribution of OT in delirium management in LTC settings.

Through the experiences provided by OTs, two key areas emerge in their clinical practice: prevention strategies and delirium screening/assessment scales and the objectives guiding therapeutic intervention. Consistent with literature [4, 9, 24], prevention and early identification of delirium, the definition of targeted therapeutic goals, and the implementation of specific interventions are the most crucial aspects in determining the OT role in managing and preventing delirium.

Regarding clinical practice, these findings indicate a predominant focus on the treatment phase of delirium in clinical practice, but further research is needed to explore how other stages, such as prevention and early detection, could be integrated into care strategies. This would enhance the overall management of delirium in LTC settings.

OTs describe interventions aimed at improving the patients' quality of life through meaningful activities, spatial–temporal orientation, sensory stimulation, and environmental modulation. Although it does not emerge clearly from our interviews, OTs also play an active role in interacting with caregivers and training care staff. The aspect of caregiver involvement, however, was not mentioned by OTs despite literature on the topic [21–23]. The preventive approach, on the other hand, appears less defined and is often implicitly considered integrated into routine OT practices. This interpretation is consistent with the previous studies [25] that highlight the difficulty of distinguishing between primary prevention and treatment in the field of delirium.

Another emerging theme is early delirium identification. OTs emphasize the importance of detecting the first signs of cognitive or behavioral changes, but they also point to a lack of systematic use of specific assessment or screening tools. This gap represents a significant barrier to timely care, especially for hypoactive forms of delirium, which are more difficult to recognize without validated tools [13, 26]. Literature recommends the routine use of scales, including the Richmond Agitation and Sedation Scale (RASS) [27], modified Richmond Agitation and Sedation Scale (mRASS) [28], 4 ‘A’s Test (4AT) [29], Delirium-O-Meter [30], Confusion Assessment Method (CAM) [31], and the Nursing Delirium Screening Scale (Nu-DESC) [32]. However, many of these tools have not been developed or validated specifically for use by OTs [33] making it difficult to standardize assessment within occupational practice and beyond.

The main barriers identified by participants are related to the lack of time, suboptimal work environment, and unclear OT role within the interdisciplinary team. The limited availability of time, which hampers the ability to implement timely and personalized interventions, emerges as a significant impediment to delivering quality care. Furthermore, the lack of adequate resources and sufficient staff in the work environment compromises the optimal management of patients with delirium [11, 13].

On the contrary, the main facilitators identified relate to the support of the interdisciplinary team and the ability to understand the expectations and wishes of the patient. Collaboration between different professionals is confirmed as a fundamental element for improving therapeutic outcomes for person with delirium, as highlighted by another research [5, 6, 34, 35].

Considering these findings, the need emerges to develop organizational frameworks that promote more structured and reflective collaboration between OTs and other professionals, enhancing their role in the prevention and treatment of delirium. This could be facilitated by shared training initiatives, interprofessional clinical discussion sessions, and supervision strategies capable of strengthening OTs’ identity awareness in geriatric-rehabilitation settings.

While offering an in-depth perspective on the role of OT in the management of delirium in LTC settings, this study presents some limitations. The credibility of the findings is linked to the specific context (LTC) and the limited number of participants [36]. Second, the dependability of the study, that is, the consistency and replicability of the research process over time, may be affected by the absence of a structured protocol for conducting interviews and analyzing data across different settings. The organizational and cultural specificities of the participants’ contexts (Italy, Spain, and Switzerland) may have significantly influenced the responses, making it difficult to transfer the results to other healthcare systems or services with different organizational models. Finally, the transferability of the findings is limited to contexts with similar characteristics to those of the services involved; therefore, it will be important to further explore these findings in future studies on a larger scale and in other healthcare systems.

## Conclusion

The findings of this qualitative study suggest that the understanding of the OTs’ role in delirium management in LTC settings by geriatricians is partial and, in many cases, fragmented. This representation appears to be influenced by a combination of factors, including limited experience in the LTC context and the absence of senior clinical figures in the teams. However, geriatricians seem to acquire a more comprehensive understanding of the OT role when they interact directly with them in their work setting, suggesting that field-based interprofessional collaboration may be a key factor in improving the perception of the OT’s contribution.

Considering these findings, it is deemed necessary to promote more structured interprofessional training initiatives that facilitate a clear and shared representation of the specific contribution of OT within care teams, even at the training level. Additionally, to optimize the coherence and effectiveness of interventions, it would be beneficial to design organizational strategies that integrate the OT in a structured way within the practices of prevention and treatment of individuals with delirium.

## Supplementary Information

Below is the link to the electronic supplementary material.

Supplementary file1 (DOCX 28 kb)
