# Supplementary material for: Geriatricians and occupational therapists’ perspectives on the role of occupational therapy in delirium care in long-term care settings: a multi-site study
Source: Eur Geriatr Med. 2025 Aug 5;16(6):1971–81. doi: 10.1007/s41999-025-01282-0 (PMC12743711; doi:10.1007/s41999-025-01282-0)
Supplement: Supplementary file 1 — Supplementary file1 (DOCX 28 kb) [file 41999_2025_1282_MOESM1_ESM.docx]

**Appendix 1 – questionnaire**

**Special Issue European Geriatric Medicine**

**Geriatricians and OT perspectives on the role of occupational therapy in Delirium Care in long-term care setting. A multi-site study**

- **First part of the interview involves the simultaneous presence of the geriatrician and the occupational therapist**

**GERIATRICIAN**

Are you a geriatric doctor? [ ] YES [ ] NO

Do you work in a LTC as a geriatrician? [ ] YES [ ] NO

In your LTC, do you have an occupational therapy service to refer to and this OTs can treat people with delirium or prevent delirium? YES [ ] NO [ ]

**OCCUPATIONAL THERAPIST**

Are you an OT? [ ] YES [ ] NO

Do you work in a LTC as a OT? [ ] YES [ ] NO

**GERIATRICIAN**

First Part: Demographic Data

- Age

- Gender

- Country

- Years of working as a consultant with geriatric expertise (number of years)

- Years of working as a consultant in the LTC unit (number of years)

- Number of OTs in the nursing home (number of OTs)

- Number of beds in the nursing home (number of beds)

- After your university education, have you attended or participated in any seminars/conferences/workshops/congresses where the main topic was occupational therapy?

**OCCUPATIONAL THERAPIST**

First Part: Demographic Data

- Age

- Gender

- Country

- Years of working as an OT (number of years)

- Years of working as an OT in geriatric settings (number of years)

- During your university education, did you have any classes related to delirium (prevent or treatment)? [ ] YES [ ] NO

- After your university education, have you attended or participated in any seminars/conferences/workshops/congresses where the main topic was delirium? [ ] YES [ ] NO

- **Second part of the interview involves geriatrician**

Reason for referral to OT (Max 3, most common)

In general, what is the main reason you request counseling/treatment in occupational therapy (in general, not only in delirium care)?

[ ] Activity of Daily Living in general

[ ] Life style in general

[ ] Recreation / Leisure in general

[ ] Activity to manage BPSD

[ ] Activity to manage Delirium

[ ] Education for the staff

[ ] Education for caregiver

[ ] Planning Discharge

[ ] Only evaluation without treatment

[ ] Cognitive retraining

[ ] Sensory integration training

Could you better explain in a few words why this choice was made?

In particular, we would like to know if the occupational therapy service is involved in the prevention of delirium or treatment of PwDelirium

- Prevention [ ] Yes [ ] No
- Treatment [ ] Yes [ ] No

What is the main role assigned to the occupational therapy service in the prevent delirium?

- Treatment (what does the occupational therapist do?)

In your role as a consultant, are you clear about the difference between the role of the occupational therapist and other health professionals?

In your opinion, what is the confidence and ability of OTs to care (prevent and treat delirium in LTC) PwDelirium? What are their resources and limitations?

Do you have suggestions for improving the quality of OT service in the geriatric setting in different European contexts?

- **Third part of the interview involves occupational therapy**

In your opinion, for the geriatrician is clear the difference between the role of occupational therapists play and the role of other health professionist (for example, they are not confused with social workers, physiotherapists, etc.)?

In your opinion, what is your confidence and ability to care (prevent and treat delirium in LTC) PwDelirium? What are their resources and limitations?

Do you have suggestions for improving the quality of OT service in the geriatric setting in different European contexts?
